# Presenteeism among nurses: An integrative review

**DOI:** 10.1016/j.ijnsa.2024.100261

**Published:** 2024-10-30

**Authors:** Maisa Gerlach, Sabine Hahn, Celine Rossier, Franziska Geese, Jan Hamers, Ramona Backhaus

**Affiliations:** a Applied Research & Development in Nursing, Bern University of Applied Sciences, Bern, CH, Switzerland; b Department of Health Services Research, Maastricht University, Maastricht, NL, Netherlands; c University Hospital Inselspital Bern, Bern, CH, Switzerland

**Keywords:** Integrative review, Presenteeism, Sickness presence, Productivity, Prevalence, Nurse

## Abstract

**Background:**

Presenteeism, a phenomenon in which employees attend work despite physical or mental limitations, is prevalent among nurses and has negative implications for patients, healthcare organizations, and nurses themselves.

**Objective:**

We aimed to present the current state of knowledge on presenteeism in nursing, focusing on prevalence rates, reasons, influencing factors, and consequences.

**Design:**

We performed an integrative review.

**Methods:**

We searched databases for studies on presenteeism in the nursing workforce published between 2018 and 2024. This review included 44 studies that met the inclusion criteria, specifically 38 quantitative studies, 4 qualitative studies, and 2 reviews.

**Results:**

The results indicated that the prevalence of nurses exhibiting symptoms of presenteeism varies between 32 % and 94 %. The influencing factors include workload, team culture, age, childcare responsibilities, job insecurity, and leadership practices. Presenteeism can lead to significant individual and organizational consequences such as increased health issues among nurses, decreased quality of patient care, and higher healthcare costs. Most studies were focused on nurses who work in hospitals, with only one study addressing nurses who work in nursing homes.

**Conclusion:**

This review highlights the high prevalence of presenteeism among nurses and its multifaceted causes and effects. This underscores the need for increased awareness and training of both nurses and management teams regarding the importance of addressing presenteeism. Further research is needed in settings such as nursing homes and outpatient care to understand the unique challenges and impacts in these environments. Efforts should focus on improving working conditions, fostering supportive organizational cultures, and implementing effective leadership practices to mitigate the negative effects of presenteeism.

**What is already known**

• Presenteeism is common among healthcare professionals, especially nurses, because of their high-stress and high-stakes roles.

• The influencing factors include workload, job insecurity, team culture, and organizational leadership.

• Presenteeism leads to negative outcomes for nurses’ health, the quality of patient care, and organizational productivity.

Alt-text: Unlabelled box

**What this paper adds**

• This study provides a comprehensive overview of the prevalence, reasons, and influencing factors of presenteeism among nurses.

• This study identifies the significant individual and organizational consequences of presenteeism, including health issues, a deterioration in the quality of patient care, and an increase in healthcare costs.

• Based on the findings, there is a need for further research in nursing homes and outpatient care settings to gain insight into the distinctive challenges and consequences. Finally, pragmatic interventions for reducing presenteeism are proposed.

Alt-text: Unlabelled box

## 1 Introduction

In today's work world, employee health is becoming increasingly important owing to the shortage of skilled labor and demographic changes, resulting in longer working hours (Helgesson et al., 2021). This increases the risk of minor complaints developing into serious illnesses and associated absences from work. Additionally, illness can decrease work performance and result in loss of productivity (Miraglia and Johns, 2016; Ospina et al., 2015). Therefore, researchers have focused on this topic to generate knowledge to support companies in improving employee health, preventing illnesses, and maintaining productivity. Research on presenteeism has been conducted in this regard. Presenteeism refers to the phenomenon of employees attending work despite physical or mental limitations that justify their absence (Freeling et al., 2020; Ruhle et al., 2019). There have been two distinct research streams in the field of presenteeism. European approach has focused on employee behavior, the reasons behind employees coming to work sick, and the subsequent consequences. In contrast, research in the United States has concentrated on the loss of productivity caused by employees coming to work sick. These two research approaches have compared research results on presenteeism between countries. Ruhle et al. (2019) agreed on a standard definition that considers productivity loss to be a consequence of presenteeism rather than presenteeism itself. This has led to research on presenteeism that examines the motives that influence individual behavior and the resulting consequences. Thus, it should contribute to a better understanding of presenteeism and make the research more comparable.

Presenteeism is a complex decision-making process in which individuals consider whether to attend work despite being ill (Lohaus and Habermann, 2019). Studies have shown that presenteeism is particularly common among employees whose work depends on others such as physicians, nurses, and teachers (Al Nuhait et al., 2017; Gustafsson Sendén et al., 2013; Martinez and Ferreira, 2012). Nursing is a high-stress, high-stakes profession that demands a constant presence owing to its direct impact on patient care and outcomes (Aiken et al., 2011, 2013; Dall'Ora et al., 2020). As a result, the prevalence rate of presenteeism may be higher than in other professions (Fischer et al., 2018; Peter et al., 2023). A meta-analysis including prevalence rates from around the world found that presenteeism has a global prevalence of 49 % among nurses (Min et al., 2022). The primary factors contributing to presenteeism among nursing staff are staff shortages, high workloads, and emotional stress (Min et al., 2022). Staff shortages result in pressure to work despite illness to avoid letting colleagues down and to maintain patient care (Dall'Ora et al., 2020). A high workload due to staff shortages, long shifts, and administrative tasks makes recovery from illness difficult (Aiken et al., 2011, 2013). Furthermore, emotional stress, burnout, and job insecurity can contribute to nurses working despite illness (Ofili et al., 2018). Presenteeism can have negative consequences for nurses, health organizations, the quality of patient care, and patient safety. The presence of nurses who are ill and should be absent can result in an increase in patient falls and medication errors, which can prolong hospital stays and lead to higher healthcare costs. Furthermore, the impact of presenteeism on organizations and staff should not be underestimated. It can exacerbate the existing health problems of the staff and negatively affect the quality of their work and job performance. Studies have also shown that presenteeism has a negative impact on private life (Li et al., 2019; Peter et al., 2023). Moreover, the impact of presenteeism on the work environment can lead to performance dissatisfaction (Rainbow et al., 2021a).

Research on presenteeism in nursing has gained importance in recent years because of its negative impact on the health of nurses and the resulting organizational consequences. This increased research effort has contributed to a better understanding of presenteeism. However, the extent and impact of presenteeism has rarely been discussed in depth. According to Freeling et al. (2020), additional research is required to examine the correlations between presenteeism, nurse well-being, patient care quality, and costs in the care sector. Since the coronavirus disease 2019 (COVID-19) outbreak, there has been growing focus on presenteeism and its impact on employees’ working conditions. Consequently, several new studies have been published in the last 5 years with a particular focus on presenteeism in healthcare and among nurses. This integrative review presents the current state of knowledge on presentism in the field of nursing care with a focus on the prevalence rate as well as the reasons and factors influencing presenteeism among nurses and describe its consequences for patients, residents, nurses, and health organizations. Accordingly, we formulated the following question for this integrative review: What are the prevalence rates, reasons, and factors related to presenteeism, and the consequences of presenteeism in nursing practice?

## 2 Methods

### 2.1 Design

An integrative methodology for the literature review was employed to address the research question and to provide an updated summary of the published evidence (Souza et al., 2010). This approach was selected because it is best suited for summarizing and analyzing multiple sources of information and produces a comprehensive understanding of a particular topic (Hopia et al., 2016; Souza et al., 2010). For this review, the Preferred Reporting Items for Systematic Reviews and Meta-Analyses (PRISMA) statement (Page et al., 2021) was followed. The Covidence Systematic Review Software was used to screen the selected publications and facilitate collaboration among researchers. This software improves communication and enforces the structure of the PRISMA framework in one system (Covidence, 2023). This integrative literature review was registered with the International Prospective Register of Systematic Reviews (PROSPERO) under the identification number CRD42023384106.

### 2.2 Search methods

Following the methodology outlined by Kable et al. (2012), the research process involved formulating a research question, selecting and documenting databases, setting search limits, establishing the inclusion and exclusion criteria (Table 1), documenting the search process, and assessing the results. The selected online databases were (1) Web of Science, (2) Cumulative Index to Nursing and Allied Health Literature (CINAHL), (3) Embase, (4) PsycINFO, and (5) PubMed. In April 2023, these databases were searched for studies on presenteeism in the nursing workforce. The most recent search to update the review with the latest published studies was conducted in April 2024. In addition, gray literature and the bibliographies of the identified studies were searched, but they did not yield any additional studies. The search criteria and specifications were applied to ensure that recent and relevant studies were related to the purpose and question of the study. For the initial search, numerous additional keywords were employed. However, this approach did not result in the identification of further relevant studies. During three meetings with a librarian, various search approaches were considered, some of which were ineffective. Therefore, the final database search included the terms Nurse [MeSH] OR “Nurs*” AND presenteeism [MeSH] OR “presenteeism” OR “sickness presence.”

**Table 1:** Inclusion criteria.

Category	Inclusion
Population	• Studies investigating presenteeism in the nursing workforce in any clinical setting • Nurses in direct care
Subject of interest	• Presenteeism (also known as sickness presenteeism or sickness presence)
Study design	• Longitudinal studies • Case-control studies • Cross-sectional studies • Qualitative studies • Reviews • Randomized controlled trials • Quasi-randomized controlled trials
Study results	• Prevalence • Influencing factors • Effects on health and long-term consequences for staff • Care of patients and residents and consequences for safety • Organization results/consequences • Cost effects
Publication language	English and German

**Fig. 1:**
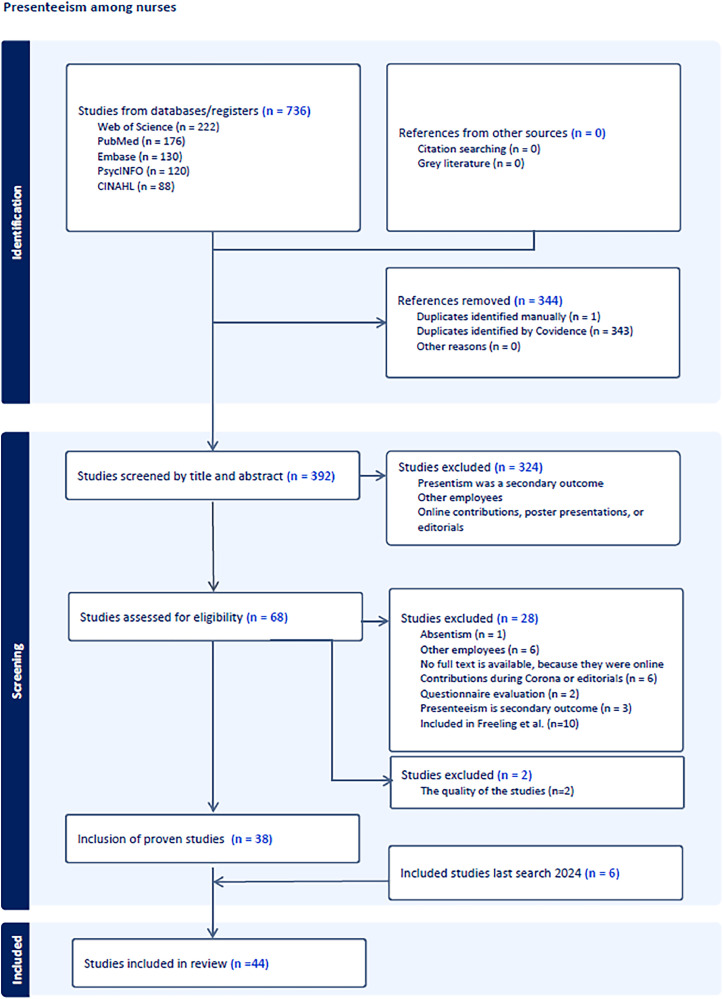
The Preferred Reporting Items for Systematic Reviews and Meta-Analyses (PRISMA) flow chart for this integrative review (adapted from Covidence systematic review software).

To ensure complete coverage and to prevent loss, no time limit was set; thus, all studies covered in the review by Freeling et al. (2020) were initially included. The first author compared studies up to 2018 with those covered by Freeling et al. (2020). Subsequently, 10 studies previously discussed by Freeling et al. (2020) were excluded (Brborović et al., 2014, 2016; Brborovi and Brborovi, 2017; Karimi et al., 2015; Kim et al., 2016; Krane et al., 2014; Letvak et al., 2012; Martinez and Ferreira, 2012; Rainbow and Steege, 2017; Schneider et al., 2018). The results of the search and screening process are shown in the PRISMA diagram in Fig. 1.

### 2.3 Screening

A total of 736 articles were identified during the search. After excluding 344 duplicate articles, the remaining 392 titles and abstracts were evaluated for relevance and adherence to the inclusion and exclusion criteria. Two reviewers independently reviewed all studies using the Covidence software (title and abstract: MG and CR; full text: MG and FG). The inclusion criteria for abstracts were that the articles had to relate specifically to the nursing workforce and that the nurses worked in direct care. Articles that did not meet these criteria were also excluded. At the full-text level, articles were excluded if they explored other types of productivity, including absenteeism and short-term disability related to absenteeism, or if the fields were not related to nursing, such as occupational health research, teaching, informal caregiving, or nurses were not included in the sample. Studies were also excluded if they analyzed questionnaires, study protocols, commentaries, case reports, or case series. After excluding the 10 studies analyzed by Freeling et al. (2020), 46 articles were considered relevant and incorporated into the literature review.

### 2.4 Quality assessment and evaluation

Specific instruments tailored to the design of each study were used to assess the quality of the 46 included studies to assess objectively the content, quality, and type of evidence available regarding presenteeism in nursing. All studies were evaluated using the checklist provided by the JBI Critical Assessment (JBI Manual for Evidence Synthesis, 2020; Munn et al., 2019). With this tool, each study was rated strong or weak, based on its overall quality (Table 2). Two reviewers (MG and CR) evaluated each study, and any discrepancies in their evaluations were discussed until a consensus was reached. Furthermore, the senior researchers (RB and SH) conducted a final review of the samples and engaged in extensive discussions regarding the inclusion and exclusion of studies. Two qualitative studies had to be excluded because the entire methodology section was unclear, and the critical appraisal questions were scored with only 3 out of 10 points. In the absence of explicit exclusion criteria for critical appraisal, this issue was discussed by the research team. As the three studies in question did not yield any new, explicitly stated results, they were excluded. The cross-sectional studies were of good quality, and all had a substantial number of participants. The statistical treatment of confounders was accurately described in the analyses, and the confounders were mostly identified but sometimes not clearly described. Unfortunately, descriptions of the inclusion criteria for the participants were sparse. Although the sample was often described, it was not explained why they were included.

**Table 2:** The influence of work-related variables on presenteeism among nurses.

Study	Workload (i.e., stress at work, working overtime, and job demands)	Emotional labor (i.e., empathy, comfort, and support for patients)	Job insecurity (i.e., reduced working hours, lack of clear career prospects, and fear of redundancy)	Shift work	Team culture (i.e., interpersonal conflict and occupational climate)	Leadership culture (i.e., lack of reward and authoritarian management style)
Baek et al. (2022)	x		x	x	x	
Banks and Pearson (2021)		x				
Durmuş et al. (2024)	x					
Fiorini et al. (2022)						x
Fan et al. (2024)						x
Freeling et al. (2020)	x	x	x	x	x	x
Gillet et al. (2020)	x	x				
Jiang et al. (2021)	x					
Kotomska et al. (2023)			x		x	
(Li et al., 2023)	x	x	x		x	
Liu et al. (2021)					x	
Liu et al. (2022)					x	
Lui et al. (2022)	x					
Min et al. (2021)				x		
Pintanela de Carvalho et al. (2021)	x					
Rainbow et al. (2020)					x	
Rainbow et al. (2021a)	x					
Santos et al. (2021)				x		
Shan et al. (2021)	x					
Shan et al. (2022)	x		x			x
Shan et al. (2023)	x					x
Song et al. (2021)	x	x				
Yi et al. (2021)		x			x	
Yoshimoto et al. (2020)				x		
Zhang et al. (2020)	x	x	x			

### 2.5 Data synthesis

A systematic synthesis was carried out to ensure a uniform presentation of the findings owing to the diversity of the studies evaluated in this review. Two researchers (MG and CR) extracted the study characteristics and data, including the design, sample, setting, and population. In addition, the influencing factors and the effects of presenteeism on care were considered, including long-term health and effects; the effects on patients; the effects on organization, including costs and productivity; and other outcomes, such as prevalence, the questionnaires used, and the influence of COVID-19. The findings of this review were clustered based on the decision-integrated model of presenteeism described by Lohaus and Habermann (2019).

## 3 Results

We included 44 studies that encompassed 37 cross-sectional studies, 1 longitudinal study, 4 qualitative studies, and 2 reviews. None of the studies met all criteria during the critical evaluation. The studies were conducted in the following countries: the United States (n = 5), China (n = 15), Hong Kong (n = 1), Brazil (n = 5), France (n = 1), Japan (n = 1), Malta (n = 1), Iran (n = 2), Portugal (n = 3), Spain (n = 1), Italy (n = 1), Korea (n = 4), Saudi Arabia (n = 1), Japan (n = 1), Australia (n = 2), Poland (n = 1), Turkey (n = 1), and Switzerland (n = 1). The two included reviews, one from the United States and one from Korea, also included international studies from Europe, the United States, Asia, and Australia. All studies, except for one that included nurses working in a nursing home, were conducted with nurses working in hospitals.

### 3.1 Prevalence of presenteeism in nursing

Nine studies reported the prevalence of presenteeism in nursing, the act of appearing sick at work (Freeling et al., 2020; Li et al., 2021; Lui et al., 2022; Min et al., 2021, 2022; Shan et al., 2023; Silva-Costa et al., 2020; Souza et al., 2010; Wu et al., 2024). Li et al. (2021) reported a prevalence rate of 70 % during the COVID-19 pandemic, whereas (Shan et al., 2023) reported a prevalence of 94 %. The remaining five studies reported a prevalence rate ranging from 33 to 85 %. Min et al. (2022) conducted a review on the prevalence of presenteeism in the nursing workforce. The estimated overall prevalence rate was 49 % (95 % confidence interval 41.1–57.4 %), with a high degree of heterogeneity (I^2^ = 98 %, p < 0.01).

### 3.2 Reasons for presenteeism in nursing

Several of the included studies described the reasons for presenteeism (Freeling et al., 2020; Laranjeira et al., 2022; Rainbow, 2019; Shan et al., 2021). The qualitative studies revealed that nurses frequently prioritize the needs of their wards and colleagues over their own health. Some nurses cannot take time off of work because they need to save their days for planned travel or to care for their children when they are sick. Some nurses experienced feelings of guilt when they were unable to fulfill their duties with their colleagues. In contrast, others experienced feelings of guilt when they were unable to fulfill their duties to their patients. Some were also afraid that because they have only a limited number of sick days or receive a lower salary, using many sick days could lead them to lose their jobs. In their quantitative study, Shan et al. (2021) found that the main reasons for presenteeism are related to the organization (51.44 %), workload (63.64 %), leave system (63.46 %), a sense of duty (59.10 %), and financial needs (52.04 %). Moreover, it is crucial to acknowledge that not all countries provide full remuneration in the event of an inability to work.

#### 3.2.1 Person-related variables

Eleven studies analyzed the personal factors related to presenteeism. The findings indicate that a number of factors—including age, gender, length of employment, marital status, parental status, and the presence of children—are associated with the practice of presenteeism among nurses. Five studies assessed the influence of age on presenteeism; four of these studies (Mosteiro‐Díaz et al., 2020; Shan et al., 2021; Shdaifat, 2023; Song et al., 2021) showed that caregivers older than 45 years are more likely to be affected by presenteeism, and one study showed that younger nurses are more likely to be affected by presenteeism (Simonetti et al., 2021). Additionally, the presence of chronic disease is a significant factor in determining whether an individual engages in presenteeism. Studies have indicated that cardiovascular diseases, musculoskeletal complaints, and mental illnesses, in particular, are associated with presenteeism. Individuals with any of these illnesses are more likely to engage in presenteeism (Min et al., 2021; Santos et al., 2021; Simonetti et al., 2021; Song et al., 2021; Sousa et al., 2023; Yi et al., 2021).

#### 3.2.2 Work-related variables

Twenty-four of the included studies discussed the influence of work-related variables on presenteeism. The researchers found that the workload (i.e., stress at work, working overtime, and job demands), emotional labor (i.e., empathy, providing comfort, and support for patients), job insecurity (i.e., reduced working hours, lack of clear career prospects, fear of redundancy, and job loss), shift work, team culture (i.e., interpersonal conflict and occupational climate), and leadership culture (i.e., lack of reward and authoritarian management style) are significant factors that influence the decision for or against presenteeism. The results of the quantitative analysis are presented in Table 2.

### 3.3 Consequences of presenteeism

Presenteeism can have a variety of consequences, including individual consequences for nursing staff and organizational consequences such as an impact on patients and productivity losses.

#### 3.3.1 Individual consequences

Ten studies investigated the consequences of presenteeism on the nursing staff and provided important findings (Fiorini et al., 2022; Freeling et al., 2020; Li et al., 2021; Pereira et al., 2022; Rainbow, 2019; Rainbow et al., 2020, 2021a; Santos et al., 2021; Zhang et al., 2020). Based on these studies, nurses evaluate presenteeism differently, according to the effects of their illness. Some view it as harmful, while others view it as beneficial. Physical health issues such as fatigue and musculoskeletal problems are closely associated with presenteeism. This can have a detrimental effect on nurses’ well-being, potentially leading to exhaustion, burnout, and malaise. Several studies have shown that presenteeism can have a negative impact on well-being, which in turn can lead to an increase in presenteeism (Freeling et al., 2020; Rainbow, 2019; Rainbow et al., 2020).

The studies found positive correlations between presenteeism, fatigue, and burnout. Moreover, presenteeism is associated with decreased health-related productivity. For example, Zhang et al. (2020) found a correlation of 0.31 between presenteeism and emotional exhaustion (p < 0.01). Simultaneously, support from superiors had a negative correlation with emotional exhaustion (r = -0.17, p < 0.01).

In the study by Fiorini et al. (2022), the participants described the effects of presenteeism on their performance and attitudes. These effects range from immediate (proximal), such as mental difficulties, to more distant (distal) consequences, which can affect the entire team. According to Rainbow (2019), the consequences of presenteeism range from work to personal and domestic problems. According to Santos et al. (2021), presenteeism exacerbates musculoskeletal complaints.

The relationship between presenteeism and perceived stress suggests that an increased risk of burnout is associated with higher presenteeism and perceived stress (Freeling et al., 2020; Rainbow, 2019; Rainbow et al., 2020). Zhang et al. (2020) reported a significant positive correlation between presenteeism and emotional exhaustion, whereas support from superiors correlated negatively with emotional exhaustion.

#### 3.3.2 Organizational consequences

Fourteen of the included studies assessed the effect of presenteeism on organizations (Fiorini et al., 2022; Freeling et al., 2020; Li et al., 2019, 2021; Lui et al., 2022; Mohammadi et al., 2021; Pereira et al., 2022; Pintanela de Carvalho et al., 2021; Rainbow, 2019; Santos et al., 2021; Zhang et al., 2024). Overall, these studies indicate that presenteeism affects efficiency and leads to a loss of work performance, which is a significant burden on organizations. Studies have also shown that presenteeism is associated with decreased quality of patient care (Guo et al., 2023). This manifests itself in higher rates of patient falls, medication errors, and impaired patient safety culture, and can depersonalize patient care (Freeling et al., 2020; Pereira et al., 2022).

The financial impact of presenteeism is significant. Depending on the study, the estimated cost per employee reporting presenteeism is between USD 14,439 and 22,237 for the organization (Freeling et al., 2020; Lui et al., 2022). Presenteeism leads to a loss of productivity and reduced work performance (Li et al., 2019, 2021; Lui et al.,2022; Santos et al., 2021; Shdaifat, 2023; Silva-Costa et al.,2020; Sousa et al., 2023). Fiorini et al. (2022) and Shan et al. (2021) reported that the average efficiency is reduced to 74.08 % when employees go sick to work. Presenteeism can negatively impact workplace dynamics, leading to team overload and interpersonal conflicts (Pereira et al., 2022; Santos et al., 2021). Turnover intentions were found to be positively correlated with presenteeism (Zhang et al., 2024).

## 4 Discussion

In this integrative review, we analyzed 44 studies to examine the prevalence, causes, related factors, and consequences of presenteeism among nurses. The results indicate that presenteeism is widespread among nurses, with a prevalence varying significantly across studies, ranging from 33 % to 94 %. All but one study were conducted in a hospital setting. The main reason for presenteeism was that nurses prioritized the team and patients over their own health and well-being. The identified factors related to presenteeism included work-related factors, such as workload and team culture, and personal factors, such as age and childcare. Presenteeism can have detrimental effects on both individual and team well-being, and productivity.

### 4.1 Personal factors

The factors related to presenteeism in nursing are personal and related to work. Personal factors, such as age, gender, having children, and health status, play a role, with older nurses and those with chronic illnesses being more affected. This finding is in line with the meta-analysis by Miraglia and Johns (2016), who found weak associations between demographic variables and presenteeism. Interestingly, studies have shown that women with children are more prone to presenteeism (Li et al., 2023; Shdaifat, 2023), perhaps because women still do most of the caring work and, therefore, often stay at home to look after their children and then go to work when they themselves are ill (Rainbow, 2019). A quantitative study supports these statements (Shdaifat, 2023). This could also explain why the female sex seems to be a predictor of higher levels of presenteeism (Silva-Costa et al., 2020; Simonetti et al., 2021). A conflict between work and privacy may arise, which could have notable effects on nurses, particularly concerning stress levels and presenteeism in the workplace (Peter et al., 2020; Peter et al., 2023).

Similarly to the review by Freeling et al. (2020), we found that nurses with a history of mental illness, psychiatric illness, and musculoskeletal disorders are more likely to engage in presenteeism. This may be because they go to work to distract themselves from their illness, or because they feel that they are missing a lot of work anyway and are therefore more likely to go to work sick out of fear (Hägerbäumer, 2017). This could also be due to the definition of presenteeism, which does not specifically define people who are chronically ill; therefore, these people naturally go to work sick more often, as they are always ill according to the definition of presenteeism.

### 4.2 Presenteeism and the team, leadership, and organizational culture

The included studies showed that work-related variables, such as stress at work, working overtime, job demands, empathy, providing comfort, support for patients, reduced working hours, lack of clear career prospects, fear of redundancy, job loss, interpersonal conflict, occupational climate, lack of reward, and authoritarian management style, influence the decision to engage in presenteeism. Notably, studies have highlighted the adverse effects of high emotional demands and a poor workplace climate on presenteeism (Shan et al., 2021; Yoshimoto et al., 2020; Zhang et al., 2020). In particular, the positive correlation between job insecurity and presenteeism emphasizes the need to improve working conditions and to create a supportive environment for nurses.

Our results demonstrate that authoritarian management styles and increased workload are potential factors that can increase the risk of presenteeism (Gillet et al., 2020; Pintanela de Carvalho et al., 2021; Shan et al., 2022). These findings suggest that revision of leadership practices can help reduce the incidence of presenteeism. Effective management and leadership are paramount in fostering a healthy and productive work culture (Northouse, 2021). Leaders must set clear goals and expectations, foster a supportive environment, and promote work–life balance (Cooper and Robertson, 2021). However, it is intriguing to consider why, despite years of discourse on leadership styles, the desired changes have not been implemented effectively. This discrepancy raises several important questions, such as why do we consistently fail to apply the well-established principles of effective leadership? One area of exploration is the gap between theoretical knowledge and practical applications. Despite extensive training programs and available resources, knowledge transfer often falls short. This can be due to resistance to change, insufficient support systems, or a lack of accountability within the organization (Alshamlani et al., 2024; Pfeffer et al., 1999; Yukl, 2013). Moreover, the existing organizational culture may not always be conducive to the adoption of new leadership styles. Entrenched habits, attitudes, and hierarchical structures can impede the implementation of progressive leadership practices (Saluy et al., 2022; Yergler, 2012). In addition to initial training, ongoing support and development of leaders are crucial for adapting and refining their approaches over time (Musooli, 2023).

Furthermore, it has become clear that an unfavorable team culture and lack of support in the workplace can also lead to an increased risk of presenteeism (Liu et al., 2021; Pereira et al., 2022; Rainbow et al., 2020, 2021b). Therefore, it is important to take steps to promote positive team dynamics and to create an environment that supports the health and well-being of nurses (Baeriswyl et al., 2016). Fostering a supportive environment within the team, in which taking the necessary time off to recuperate is encouraged, can effectively mitigate presenteeism (Gillet et al., 2020). This notion is emphasized by the significant influence of organizational culture on presenteeism, particularly when there is a prevailing attitude that prioritizes long working hours and a constant presence in the workplace (Webster et al., 2019). Such a culture can exert pressure on employees to attend work, even when they are ill, because they may worry that being absent will be perceived as a lack of dedication or commitment to their roles. Consequently, employees may feel obligated to work despite their health condition in such a setting. These findings underscore the importance of addressing both individual and work-related factors to mitigate presenteeism among nurses (Rainbow et al., 2019).

### 4.3 Implications for practice

Comprehensive strategies should be implemented to address the variables related to presenteeism. This includes efforts to lessen the emotional burden experienced by nurses, possibly through increased access to counseling services or support groups (Kotomska et al., 2023). Reducing workload can be achieved through optimizing staffing levels, providing adequate resources, and streamlining administrative tasks (Fan et al., 2024). Improving corporate culture entails fostering an environment in which open communication, mutual support, and an understanding of presenteeism are encouraged among team members and leadership (Alilyyani, 2018). Introducing new working models, such as flexible schedules or remote work options, can contribute to better work–life balance for nurses, thus alleviating stress and preventing presenteeism (Allen, 2013). Additionally, advocating for improved working conditions in the care sector, such as better pay, benefits, and job security, is crucial for promoting overall well-being and reducing the prevalence of presenteeism among nurses (Aiken, 2002, McHugh, 2014).

### 4.4 Further investigation

Only one study investigated presenteeism among nurses who work in nursing homes. This is a remarkable phenomenon, particularly in nursing homes, given the vulnerability of the residents, the demanding nature of caregiving, the significant long-term consequences on the health of both nurses and residents, and the acute shortage of skilled workers. Therefore, future research should focus on determining the prevalence of presenteeism in nursing homes or outpatient care, identifying the factors influencing its occurrence, and analyzing its long-term effects on the health of nursing staff and patients.

Strategies should be developed to support nurses in dealing with presenteeism. This should include the provision of support systems and resources aimed at minimizing the negative impacts of presenteeism, such as the establishment of stress management programs specifically for caregivers. Further research should concentrate on approaches designed to prevent the phenomenon of presenteeism.

Furthermore, it is necessary to examine how presenteeism manifests in cultural and linguistic contexts. Determining whether and how cultural differences influence the emergence and management of presenteeism is important. Such research approaches will enable us to gain a deeper understanding of presenteeism in the care sector, to develop effective strategies to address it, and to learn from countries in which presenteeism is less prevalent.

## 5 Limitations

We made efforts to develop more comprehensive search strategies. However, our review still had limitations, such as the potential oversight of relevant studies due to keyword selection or search criteria. The exclusion of certain productivity measures and fields not directly related to nursing could overlook valuable insights. Language bias may have occurred because we focused on English and German publications. Although we used the JBI Critical Assessment Tool to evaluate the quality of the included studies, it is important to note that subjective elements and reviewer discrepancies could introduce bias. Additionally, relying solely on published articles may lead to a publication bias and limited generalizability of the findings. Despite attempts at systematic synthesis, the diversity of study designs and outcomes poses challenges to presenting uniform results. Addressing these limitations can enhance the transparency and reliability of research.

## 6 Conclusions

In this integrative review, we have provided an overview of the current state of research on presenteeism in nursing. Personal factors as well as the management, team, and organizational culture are important factors in the decision for or against presenteeism. Therefore, the implication for practice is to sensitize nurses to this issue and to provide additional training to managers on the importance of presenteeism. Furthermore, it is necessary to examine the leadership culture in its entirety. This includes understanding why the knowledge acquired through training is not being implemented or why it does not reach the nurses. Additionally, identifying the challenges associated with implementing a positive leadership culture is essential. However, most studies come from the hospital sector, which makes it difficult to generalize them to the nursing profession. Therefore, the results of this study emphasize the need for further research on the phenomenon of presenteeism in nursing. To gain a more comprehensive understanding of this problem in other settings, it would be beneficial to examine the contexts of nursing homes and outpatient care. The complexity of care and the lack of adequately trained staff in this setting can result in an increased demand for nurses.

## Funding sources

This review received no external funding.

## Declaration of using AI

During the preparation of this work, the authors used ChatGPT to improve the English in the text. After using this tool, the authors reviewed the content, made edits as needed, and take full responsibility for the content of the publication.

## CRediT authorship contribution statement

**Maisa Gerlach:** Writing – original draft, Visualization, Validation, Software, Resources, Project administration, Methodology, Investigation, Formal analysis, Data curation, Conceptualization. **Sabine Hahn:** Writing – review & editing, Supervision, Methodology, Conceptualization. **Celine Rossier:** Resources, Formal analysis, Data curation. **Franziska Geese:** Resources, Formal analysis. **Jan Hamers:** Writing – review & editing, Supervision. **Ramona Backhaus:** Writing – review & editing, Writing – original draft, Supervision, Resources, Project administration, Methodology.

## Declaration of competing interest

The authors declare that they have no conflicts of interest.

## Figures and Tables

**Table:** 

**Study**	**Q1**	**Q2**	**Q3**	**Q4**	**Q5**	**Q6**	**Q7**	**Q8**
Baek et al. (2022)	U	U	Y	Y	Y	Y	Y	Y
Banks and Pearson (2021)	U	Y	Y	Y	Y	Y	Y	Y
Durmuş et al. (2024)	Y	Y	Y	Y	U	U	Y	Y
Fan et al. (2024)	Y	Y	Y	Y	Y	Y	Y	Y
Fiorini et al. (2022)	U	Y	Y	Y	Y	Y	Y	Y
Gillet et al. (2020)	U	Y	Y	Y	Y	Y	Y	Y
Guo et al. (2023)	Y	Y	Y	Y	Y	Y	Y	Y
Jiang et al. (2021)	U	Y	Y	Y	Y	Y	Y	Y
Kotomska et al. (2023)	Y	Y	Y	Y	Y	Y	Y	Y
Li et al. (2019)	U	Y	Y	Y	Y	Y	Y	Y
Li et al. (2021)	U	Y	Y	Y	Y	Y	Y	Y
Li et al. (2023)	Y	Y	Y	Y	Y	Y	Y	Y
Liu et al. (2021)	U	Y	Y	Y	Y	Y	Y	Y
Liu et al. (2022)	U	Y	Y	Y	Y	Y	Y	Y
Lui et al. (2022)	U	Y	Y	Y	Y	Y	Y	Y
Min et al. (2021)	U	Y	Y	Y	Y	Y	Y	Y
Mosteiro‐Díaz et al. (2020)	Y	Y	Y	Y	Y	Y	Y	Y
Pintanela de Carvalho et al. (2021)	Y	Y	Y	Y	U	U	Y	Y
Rainbow et al. (2020)	U	Y	Y	Y	Y	Y	Y	Y
Rainbow et al. (2021)	Y	Y	Y	Y	U	U	Y	Y
Santos et al. (2021)	Y	Y	Y	Y	Y	Y	Y	Y
Shan et al. (2021)	Y	Y	Y	Y	Y	Y	Y	Y
Shan et al. (2022)	Y	Y	Y	Y	Y	Y	Y	Y
Shan et al. (2023)	Y	Y	Y	Y	Y	Y	Y	Y
Shdaifat (2023)	Y	Y	Y	Y	Y	Y	Y	Y
Silva-Costa et al. (2020)	Y	Y	Y	Y	Y	Y	Y	Y
Simonetti et al. (2021)	Y	Y	Y	Y	Y	Y	Y	Y
Song et al. (2021)	U	Y	Y	Y	Y	Y	Y	Y
Sousa et al. (2023)	Y	Y	Y	Y	Y	Y	Y	Y
Wu et al. (2024)	Y	Y	Y	Y	Y	Y	Y	Y
Yi et al. (2021)	U	Y	Y	Y	Y	Y	Y	Y
Yoshimoto et al. (2020)	U	Y	Y	Y	Y	Y	Y	Y
Zhang et al. (2020)	Y	Y	Y	Y	Y	Y	Y	Y
Zhang et al. (2024)	Y	Y	Y	Y	Y	Y	Y	Y

Abbreviations—Y = yes; N = no; U = unclear; NA = not applicable.

Questions from the JBI Critical Appraisal for Cross-Sectional Studies—Q1: Were the criteria for inclusion in the sample clearly defined?; Q2: Were the study subjects and the setting described in detail?; Q3: Was the exposure measured in a valid and reliable way?; Q4: Were objective, standard criteria used for measurement of the condition?; Q5: Were confounding factors identified?; Q6: Were strategies to deal with confounding factors stated?; Q7: Were the outcomes measured in a valid and reliable way?; Q8: Was appropriate statistical analysis used?

**Table:** 

**Study**	**Q1**	**Q2**	**Q3**	**Q4**	**Q5**	**Q6**	**Q7**	**Q8**	**Q9**	**Q10**
Mohammadi et al. (2021)	Y	Y	Y	Y	Y	N	N	Y	Y	Y
Mohammadi et al. (2021)	Y	Y	Y	Y	Y	N	N	Y	Y	Y
Pereira et al. (2022)	Y	Y	Y	Y	Y	Y	Y	Y	Y	Y
Rainbow (2019)	Y	Y	Y	Y	Y	Y	U	Y	U	Y
Rainbow et al. (2021**)**	U	U	N	Y	Y	U	N	Y	Y	Y
Laranjeira et al. (2022)	Y	Y	Y	Y	Y	U	U	Y	Y	Y

Abbreviations—Y = yes; N = no; U = unclear; NA = not applicable.

Questions from the JBI Critical Appraisal for Qualitative Studies—Q1: Is there congruity between the stated philosophical perspective and the research methodology?; Q2: Is there congruity between the research methodology and the research question or objectives?; Q3: Is there congruity between the research methodology and the methods used to collect data?; Q4: Is there congruity between the research methodology and the representation and analysis of data?; Q5: Is there congruity between the research methodology and the interpretation of results?; Q6: Is there a statement locating the researcher culturally or theoretically?; Q7: Is the influence of the researcher on the research, and vice- versa, addressed?; Q8: Are participants, and their voices, adequately represented?; Q9: Is the research ethical according to current criteria or, for recent studies, and is there evidence of ethical approval by an appropriate body?; Q9: Was appropriate statistical analysis used?; Q10: Do the conclusions drawn in the research report flow from the analysis, or interpretation, of the data?

**Table:** 

**Study**	**Q1**	**Q2**	**Q3**	**Q4**	**Q5**	**Q6**	**Q7**	**Q8**	**Q9**	**Q10**	**Q11**
Cardoso dos Santos et al. (2018)	Y	Y	Y	Y	Y	**N**	**Y**	**Y**	**Y**	**Y**	**Y**

Abbreviations—Y = yes; N = no; U = unclear; NA = not applicable.

Questions from the JBI Critical Appraisal for Cohort Studies—Q1: Were the two groups similar and recruited from the same population?; Q2: Were the exposures measured similarly to assign people to both exposed and unexposed groups?; Q3:Was the exposure measured in a valid and reliable way?; Q4: Were confounding factors identified?; Q5: Were strategies to deal with confounding factors stated?; Q6: Were the groups/participants free of the outcome at the start of the study (or at the moment of exposure)?; Q7: Were the outcomes measured in a valid and reliable way?; Q8: Was the follow up time reported and sufficient to be long enough for outcomes to occur?; Q9: Was follow-up complete, and if not, were the reasons to loss to follow up described and explored?; Q10: Were strategies to address incomplete follow up utilized?; Q11: Was appropriate statistical analysis used?

**Table:** 

Study	Q1	Q2	Q3	Q4	Q5	Q6	Q7	Q8	Q9	Q10	Q11
Freeling et al. (2020)	Y	Y	Y	Y	Y	Y	Y	U	Y	Y	Y
Min et al. (2022)	Y	Y	Y	Y	Y	Y	Y	Y	Y	Y	Y

Abbreviations—Y = yes; N = no; U = unclear; NA = not applicable.

Questions from the JBI Critical Appraisal for Reviews—Q1: Is the review question clearly and explicitly stated?; Q2: Were the inclusion criteria appropriate for the review question?; Q3: Was the search strategy appropriate?; Q4: Were the sources and resources used to search for studies adequate?; Q5: Were the criteria for appraising studies appropriate?; Q6; Was critical appraisal conducted by two or more reviewers independently?; Q7: Were there methods to minimize errors in data extraction?; Q8: Were the methods used to combine studies appropriate?; Q9: Was the likelihood of publication bias assessed?; Q10: Were recommendations for policy and/or practice supported by the reported data?; Q11: Were the specific directives for new research appropriate?

**Table:** 

Author, year, title, and country	Study aim	Methods	Sample and setting	Key findings
Baek et al. (2022) Relationship between occupational stress, sleep disturbance, and presenteeism of shiftwork nurses South Korea	To determine the relationships between occupational stress, sleep disturbance, and presenteeism in shiftwork nurses in South Korea	Cross-sectional study	442 shiftwork nurses working in hospitals	**Influencing factors** • Association between subscales of occupational stress and presenteeism In multiple regression analyses, job insecurity, interpersonal conflict, occupational climate, lack of reward, and job demand were significantly related to presenteeism. • In multiple regression analyses, job insecurity (β = 0.19, p < 0.001), interpersonal conflict (β = 0.13, p = 0.013), occupational climate (β = 0.13, p = 0.015), lack of reward (β = 0.13, p = 0.021), and job demand (β = 0.10, p = 0.034) were significantly related to presenteeism. • The authors analyzed the role of sleep disturbance in moderating the relationship between occupational stress and presenteeism to understand how occupational stress can impact presenteeism. • In Step 1, occupational stress and sleep disturbance were significantly related to presenteeism. • In Step 2, the interaction of occupational stress and sleep disturbance had a positive relationship with presenteeism. • The strength of the relationship between occupational stress and presenteeism changed with sleep disturbance.
Banks and Pearson (2021) Personality, staff attitudes and their association with absenteeism and presenteeism in Australian public sector hospital-based nurses: A cross-sectional study Australia	To examine how personality and attitudes to sick leave influence nurses’ self-reported rates of absenteeism and presenteeism	Cross sectional study	320 nurses	**Influencing factors** • Presenteeism was significantly associated with younger age, higher reported stress, lower emotional stability, and disagreeing that a culture of entitlement to sick leave exists. • Conscientiousness was significantly associated with a reduced likelihood of being absent from work as was emotional stability. • Predictors of presenteeism in the final model included emotional stability and stress. • Overall, of the 12 % variance in presenteeism, the personality measures explained 3.3 %; age, sex, work pattern, and stress explained 6.4 %; and attitudes explained 2.3 %.
Cardoso dos Santos et al. (2018) Presenteeism and musculoskeletal symptoms among nursing professionals. Brazil	To investigate the relationship between musculoskeletal symptoms and presenteeism	Longitudinal Study	211 nurses working in hospitals	**Influencing factors**The regression analysis focusing on presenteeism and pain in various anatomical regions revealed the following • **Shoulder pain analysis** ○ Among the 81 participants observed, male nursing assistants had a mean presenteeism score of 22.2096. ○ Female nursing professionals scored 1.5424 points lower, while nursing technicians scored 0.8176 points higher and nurses scored 1.6143 points higher. ○ Each year of age was associated with a 0.0704 increase in the presenteeism score. ○ Nursing professionals reporting shoulder pain had a 1.5830 decrease in presenteeism score. • **Elbow pain analysis** ○ Among the 27 participants observed, male nursing assistants had a mean presenteeism score of 20.8536. ○ Women scored 2.1827 points higher, while nursing technicians scored 0.7063 points lower and nurses scored 14.2367 points higher. ○ Each year of age was associated with a 0.0753 decrease in presenteeism score. ○ Nursing professionals reporting elbow pain had a 1.0330 increase in presenteeism score. • **Wrist and/or hand pain analysis** ○ Among the 62 participants observed, male nursing assistants had a mean presenteeism score of 22.4353. ○ Women scored 1.6264 points lower, while nursing technicians scored 2.8956 points higher and nurses scored 1.0873 points higher. ○ Each year of age was associated with a 0.0205 increase in presenteeism score. ○ Nursing professionals reporting wrist and/or hand pain had a 0.6074 increase in presenteeism score. • **Knee pain analysis** ○ Among the 74 participants observed, male nursing assistants had a mean presenteeism score of 21.9099. ○ Women scored 3.1231 points lower, while nursing technicians scored 1.7219 points lower and nurses scored 0.164 points higher. ○ Each year of age was associated with a 0.0828 increase in presenteeism score. ○ Nursing professionals reporting knee pain had a 1.0125 increase in presenteeism score. Overall, there were no significant differences observed in presenteeism scores concerning pain across the anatomical regions analyzed.
Durmuş et al. (2024) The effect of nurses' perceived workplace incivility on their presenteeism and turnover intention: The mediating role of work stress and psychological resilience Turkey	To determine the effects of nurses’ perceived workplace incivility on nurses’ presenteeism and turnover intention and to reveal the mediating role of work stress and psychological resilience in the possible impact	Cross-sectional study	302 nurses working in direct patient care	**Effect of workplace incivility** • On turnover intention: positive and significant effect (β = 0.409, t = 8.645, p < 0.001). • On presenteeism: positive and significant effect (β = 0.374, t = 6.719, p < 0.001). • On work stress: positive and significant effect (β = 0.498, t = 12.028, p < 0.001). • On psychological resilience: negative and significant effect (β = -0.321, t = 6.204, p < 0.001). **Effect of psychological resilience** • On presenteeism: negative and significant effect (β = -0.249, t = 4.916, p < 0.001). • On turnover intention: no significant effect (β = -0.099, t = 1.556, p = 0.120). **Effect of work stress** • On turnover intention: positive and significant effect (β = 0.330, t = 5.072, p < 0.001). • On presenteeism: positive and significant effect (β = 0.297, t = 5.467, p < 0.001).
Fan et al. (2024) The role of the nursing work environment, head nurse leadership and presenteeism in job embeddedness among new nurses: A cross-sectional multicentre study China	To reveal how the nursing work environment, head nurse leadership, and presenteeism shape job embeddedness in this population from the perspective of conservation of resources theory	Cross-sectional study	436 nurses	Presenteeism (r = -0.349, p < 0.01), hospital grade (r = -0.112, p < 0.05), weekly working hours (r = -0.160, p < 0.05), and the number of night shifts per week (r = -0.100, p < 0.05) correlated negatively with job embeddedness. The nursing work environment correlated positively with head nurse leadership (r = 0.753, p < 0.01) and negatively with presenteeism (r=-0.301, p < 0.01). There was a negative correlation between head nurse leadership and presenteeism (r = -0.335, p < 0.01).
Fiorini et al. (2022) Nurses’ perceived work performance and health during presenteeism: Cross-sectional associations with personal and organisational factors Malta	To determine personal and organizational factors associated with work performance and illness outcomes during presenteeism in a cohort of nurses	Cross-sectional study	270 nurses working with older adults	**Influencing factors** • Hierarchical multiple linear regression revealed that age (β = 0.18, p = 0.008) was the only demographic variable to contribute to the final model. Less negative perceptions of illness were also associated with better performance (β = -0.16, p = 0.008). Finally, better performance was linked with greater engagement (β = 0.18, p = 0.004) and support from supervisors (β = 0.13, p = 0.033). The final model explained 14 % of the variance (F(6, 263) = 8.23, p < 0.001). **Organizational consequences** • The mean Stanford Presenteeism Scale (SPS-6) score (Stanford Presenteeism Scale) was 17.50 (standard deviation 4.22). • Bivariate correlations revealed that variables within all three-factor categories were significantly associated with perceived performance loss during presenteeism. **Consequences for nurses** • Four participants rated presenteeism as very beneficial, 8 as beneficial, 56 as having no effect on illness, 187 as harmful, and 15 as very harmful.
Freeling et al. (2020) Painting a picture of nurse presenteeism: A multi-country integrative review United States (also included international studies)	To conduct a review of the impact of presenteeism as it relates to the nursing workforce	Integrative review		**Influencing factors** • As expected, the results consistently showed that participants with preexisting mental, physical, and chronic health conditions had higher rates of presenteeism. • Mental health conditions linked to presenteeism included anxiety and depression. • Depression had the highest prevalence and strongest association with presenteeism. **Reasons for presenteeism** • The participants described that the reason for their presenteeism was the belief of having to fulfill their duty to work, regardless of sickness. **Consequences for nurses** • The physical health conditions strongly associated with presenteeism were fatigue, musculoskeletal issues, and cold/allergy symptoms. • Several studies found that presenteeism is associated with risks to nursing well-being through exhaustion and burnout, stress, and general well-being. • Some studies found that presenteeism can decrease well-being, which, in turn, can increase presenteeism. **Consequences for patients**Several studies showed a link between presenteeism among nurses and a decline in patient care.Specifically, presenteeism was associated with higher rates of patient falls, medication errors, and patient safety culture.Interestingly, Brborovic and Brborovic (2017) found that patient safety culture shapes absenteeism and presenteeism: Participants with higher patient safety culture (PSC) scores are more likely to exhibit presenteeism, while people with lower PSC scores are more likely to exhibit absenteeism.**Organizational consequences** • There was a loss of USD 22,237 per employee who reported presenteeism in a sample of pharmacists and registered nurses and USD 14,439 per nurse. • The authors also estimated the costs concerning nurse presenteeism and the relationship to patient falls and medication errors.
Gillet et al. (2020) The effects of job demands on nurses' burnout and presenteeism through sleep quality and relaxation France	To investigate the indirect effects of emotional dissonance and workload on presenteeism and emotional exhaustion through sleep quality and relaxation	Cross-sectional study	652 nurses, and 104 nursing assistants working in hospitals	**Influencing factors** • Correlation analyses showed significant relationships between workload and respectively sleep quality (r = -0.257, p < 0.0 01), relaxation (r = -0.187, p < 0.01), emotional exhaustion (r = 0.478, p < 0.001), and presenteeism (r = 0.189, p < 0.01); between emotional dissonance and sleep quality (r = -0.328, p < 0.001), relaxation (r = -0.192, p < 0.01), emotional exhaustion (r = 0.422, p < 0.001), and presenteeism (r = 0.257, p < 0.001); and between sleep quality and emotional exhaustion (r = -0.536, p < 0.001) and presenteeism (r = -0.373, p < 0.001).
Guo et al. (2023) The relationship between presenteeism among nurses and patients’ experience in tertiary hospitals in China China	To observe the current situation of nurses ’ presenteeism and the relationship between presenteeism among nurses and patient perceptions and to examine its implications for nursing management	Cross-sectional study	433 nurses and 435 patients	There was a negative correlation between nurse presenteeism and patient experience, that is, the higher the nurse presenteeism, the worse the patient experience (r = 0.12, p < 0.05). Presenteeism among nurses correlated negatively with patient experience in environmental logistics and technical quality (r = 0.13 and 0.11, respectively, p < 0.05 for both)
Jiang et al. (2021) Nurses’ occupational stress and presenteeism: The mediating role of public service motivation and the moderating role of health China	To investigate the links between stress at work, public service motivation, health, and presenteeism.	Cross-sectional study	981 nurses working in hospitals	**Influencing factors** • The equation showed that challenge stress had a negative effect on public service motivation, that challenge stress was significantly positively related to presenteeism, and that public service motivation correlated negatively with presenteeism. • The interaction between challenge stress and health had a significantly negative predictive effect on presenteeism, suggesting that the mediating effect of public service motivation on the relationship between challenge stress and presenteeism tended to decrease with the improvement of health.
Kotomska et al. (2023) Presenteeism – A common phenomenon in the study population of nurses Poland	To indicate the predictors of operating room nurses’ presence at work despite disease symptoms and classification of the symptoms	Cross-sectional study	861 working nurses	Numerous factors contributed significantly to the attitude of presenteeism: a sense of responsibility toward workmates (p = 0.000, χ^2^ = 16.86) and the employer (p = 0.000, χ^2^ = 14.49) or concerns about stable employment (p = 0.016, χ^2^ = 5.89). Interestingly, there was a lack of statistical significance for the sense of responsibility toward the patient. Moreover, the respondents were aware that coming to work when ill affects the quality of work and contributes to a higher risk of committing an error.
Laranjeira et al. (2022) Contributing factors of presenteeism among Portuguese and Swiss nurses: A qualitative study using focus groups Portugal and Switzerland	To explore the factors associated with presenteeism among frontline nurses and nurse managers in acute, primary, and long-term healthcare settings in Portugal and Switzerland	Qualitative research	55 nurses working in different healthcare settings	**Reasons for presenteeism** • Unfamiliar terminology • The paradoxical effect of “being present” but absent. ○ Struggle between knowing and doing ○ Struggle between the collective good (colleagues and patients) and self-sacrifice • Presenteeism as a survival strategy ○ Presenteeism as a short-term strategy ○ Presenteeism as an “indispensable element” for functioning teams **Influencing factors** • Institutional disinterest toward employees ○ Workload ○ Lack of investment ○ Lack of support from specialized services • Paradigm shift: the tension between person-centered and task-centered care • Sudden changes in care practices due to the COVID-19 pandemic • Lack of shared work perspectives with hierarchical superiors • Financial burden of being absent from work • Misfit of human responses ○ Variability in individual responses ○ Manifestations of presenteeism are devalued. ○ Lack of personal resources Fear of the consequences of absenteeism
Li et al. (2019) The effect of presenteeism on productivity loss in nurses: The mediation of health and the moderation of general self-efficacy China	To explore the current situation of presenteeism among Chinese nurses and the mediating effect of health and the moderating effect of general self-efficacy between presenteeism and productivity loss	Cross-sectional study	340 female nurses working in hospitals	**Organizational consequences** • Presenteeism was significantly associated with health and productivity loss, health was significantly associated with productivity loss, and general self-efficacy was negatively associated with productivity loss.
Li et al. (2021) Serial-multiple mediation of job burnout and fatigue in the relationship between sickness presenteeism and productivity loss in nurses: A multicenter cross-sectional study China	To investigate the serial, multiple mediating effect of job burnout and fatigue in the relationship between sickness presenteeism and productivity loss among nurses	Cross-sectional study	2968 nurses working in hospitals	**Prevalence of presenteeism** • Sickness presenteeism exhibited a prevalence of 70.6 % during the COVID-19 pandemic. **Organizational consequences** • The average score of health-related productivity loss was 15.05 ± 4.52. **Consequences for nurses** • Sickness presenteeism correlated positively with fatigue and job burnout, while job burnout correlated positively with nurse fatigue. Sickness presenteeism, fatigue, and job burnout correlated positively with health-related productivity loss.
Li et al. (2023) Latent profile analysis and influence factors study of presenteeism among ICU nurses in China China	To investigate the subtypes of presenteeism among nurses working in intensive care units	Cross-sectional study	509 nurses working in intensive care units	The model comprised three profiles: the low presenteeism–normal coping group (18.3 %), the moderate presenteeism group (47.9 %), and the high presenteeism–work limitation group (33.8 %). Multiple logistic regression showed that secondary hospitals (odds ratio = 0.116, p = 0.015), good physical health (odds ratio = 0.084, p = 0.023), general physical health (odds ratio = 0.016, p = 0.037), intensive care unit human resource allocation of 1:2.5–3 (odds ratio = 0.315, p = 0.007), and higher social support scores (odds ratio = 0.975, p = 0.047) were more likely to be grouped into the low presenteeism–normal coping group; married with no children (odds ratio = 24.554, p = 0.005) was more likely to be grouped into the moderate presenteeism group; and having experienced workplace violence in the past year (odds ratio = 1.182, p = 0.049) was more likely to be grouped into high presenteeism–work limitation group.
Liu et al. (2021) The influence of team mindfulness on nurses' presenteeism: A cross-sectional study from the perspective of sensemaking China	To explore the influence of team mindfulness on nurses' presenteeism and to assess the mediation effect of perceived empathic concern and perceived insider status and the moderating effect of organizational formalization	Cross-sectional study	396 nurses working in hospitals	**Influencing factors** • Team mindfulness exerted a significant negative effect on nurses’ presenteeism (β = -0.081, p < 0.05) • Team mindfulness had a significant positive effect on perceived insider status (β = 0.053, p < 0.053) • Insider status exerted a significant negative effect on nurses’ presenteeism (β = -0.085) • Team mindfulness and organizational formalization significantly affected perceived empathic concern.
Liu et al. (2022) Job crafting and nurses' presenteeism: The effects of job embeddedness and job irreplaceability China	To explore the relationship between job crafting and nurses’ presenteeism from the perspective of the individual internal initiative	Cross-sectional study	900 nurses working in hospitals	**Influencing factors** • Job crafting correlated positively with job embeddedness, and job embeddedness correlated negatively with presenteeism. • The main effects of job embeddedness and job irreplaceability on presenteeism were significant, and the interaction between the two significantly impacted presenteeism. • With increasing job irreplaceability, nurses with lower job embeddedness experienced more presenteeism than the nurses with higher job embeddedness.
Lui et al. (2022) Does seasonal influenza related hospital occupancy surge impact hospital staff sickness presenteeism and productivity costs? Hong Kong	To quantify how seasonal influenza hospital occupancy surge impacts nurses’ sickness presenteeism and related productivity costs in high-intensity inpatient metropolitan hospitals	Cross-sectional study	824 nurses working at hospital 1, 835 nurses working at hospital 2, and 1379 nurses working at hospital 3	**Prevalence of presenteeism** • The prevalence of presenteeism was 71.3 % in the sampled nurses (based on ≥2 presenteeism events in the past year). The mean self-rated productivity score of the nurses was 6.67 out of 10. **Organizational consequences** • Generalized estimating equation regression results showed that a 6.8 % increase (1 standard deviation) in inpatient occupancy rate was associated with a 19 % increase (odds ratio = 1.19, 95 % confidence interval 1.05–1.35) in presenteeism (≥2 events in past year) • Nurses who took sick leave reported a productivity loss of USD 2081 per nurse (including on-the-job productivity loss costs and sick leave costs). On-the-job productivity losses were not significantly different between those who worked healthy (USD 1983) and exhibited presenteeism (≥2 presenteeism events in past year; USD 2008).
Min et al. (2021) Sickness presenteeism in shift and non-shift nurses: Using the Fifth Korean Working Conditions Survey Korea	To determine the prevalence of sickness presenteeism and to examine the associations among working conditions, health status, and SP among shift and non-shift nurses in South Korean hospitals	Cross-sectional study	272 nurses working in hospitals	**Prevalence of presenteeism** • 21.8 % reported experiencing sickness presenteeism in the preceding 12 months. **Influencing factors** • The rate of sickness presenteeism was higher among shift nurses (n = 35, weighted percentage 54.6 %) than non-shift nurses (n = 28, weighted percentage 45.4 %). • Among shift nurses, only a quick return, sleep disturbance, and health problems were significantly associated with sickness presenteeism • The sickness presenteeism rate was higher among nurses who experienced a quick return (54.2 %) than those who did not (18.9 %). • Nurses who experienced sleep disturbance experienced more sickness presenteeism (36.9 %) than those who did not (16.7 %). • Additionally, sickness presenteeism was higher among those with health problems (36.9 %) than among those without (12.6 %). Among non-shift nurses, only total working hours and health problems were significantly associated with sickness presenteeism. • The sickness presenteeism rate was the highest among nurses who worked ≥53 h per week. Nurses who experienced health problems experienced more sickness presenteeism (38.3 %) than those who did not (16.4 %).
Min et al. (2022) Global prevalence of presenteeism in the nursing workforce: A meta-analysis of 28 studies from 14 countries. Korea (also included international studies)	To determine the pooled prevalence of presenteeism in the nursing workforce in various countries and clinical settings	Meta-analysis		**Global prevalence** • The estimated overall pooled prevalence of presenteeism in the nursing workforce was 49.2 % (95 % confidence interval 41.1 %–57.4 %) in a random-effects model. There was high heterogeneity (I^2^ = 98 %, p < 0.01).
Mohammadi et al. (2021) Exploring the concept of presenteeism in nursing: A hybrid concept analysis Iran	To explore the concept of presenteeism in nursing using a hybrid concept analysis method	Review and qualitative interviews	17 nurses	• The findings suggest that presenteeism in nursing can be considered as a kind of fall from balance, involving the non-actualization of capacities in both physical and emotional aspects. **Organizational consequences** • It was revealed that presenteeism reduces nursing performance and affects patient care, including decreased efficiency, job satisfaction, and increased human error.
Mohammadi et al. (2021) The nurse without a nurse: The antecedents of presenteeism in nursing Iran	To explain the reasons for presenteeism in nurses	Qualitative research	17 nurses working in hospitals	**Influencing factors** • Perceived injustice by nurses led to the emergence of presenteeism: Nurses suffer emotionally in an environment full of injustice. • There is no justice in giving a chance to leave and rest. • Organizational policies are forced to impose a kind of obligatory presence on the nurse due to problems such as lack of nursing staff. • Organizational policies are forced to impose a kind of obligatory presence on the nurse due to problems such as lack of nursing staff. • A participant mentioned the lack of trust and confidence in nurses. • The participants discussed the mistaken support of managers who follow organizational policies that encourage presenteeism. • According to the participants' experiences, allergies and runny nose, asthma, migraine, food poisoning, diarrhea, vomiting, shoulder pain, knee pain, neck disc, headache, neck pain, toothache, and chronic diseases were among the diseases that could lead to presenteeism.
Mosteiro‐Díaz et al. (2020) Presenteeism in nurses: comparative study of Spanish, Portuguese and Brazilian nurses Portugal, Spain, and Brazil	To compare presenteeism levels among three samples of nurses and to identify the relationship between presenteeism and sociodemographic and professional characteristics	Cross-sectional study	659 nurses working in hospitals	**Influencing factors** • Age and professional experience were significant predictors of total presenteeism, but only professional experience maintained its significance in the adjusted model. • Younger nurses with less professional experience presented lower levels of presenteeism but greater psychological commitment
Pereira et al. (2022) Consequences of nurse presenteeism in Switzerland and Portugal and strategies to minimize it: A qualitative study Switzerland and Portugal	To explore the cross-cultural aspects of the perceptions, attitudes, and experiences of nurses in Portugal and Switzerland about (a) the consequences of presenteeism, and (b) strategies for minimizing it in different acute care, nursing home, and community healthcare settings	Qualitative research	55 nurses working in acute care, nursing home, and community healthcare settings	**Influencing factors** • Leadership • Team culture **Consequences for nurses** • Nurses’ health and well-being ○ The physical, psychological, and mental impact Self-medication to keep working • Frustration and job dissatisfaction • Family/social relationships ○ Failing capacity to manage relationships. ○ Committed family dynamics **Organizational consequences** • Workplace dynamics ○ Decrease in productivity, team overload, peer “contagion” effect, and interpersonal conflicts with peers and patients **Consequences for patients** • Quality of care and patient safety ○ Depersonalization of care ○ Compromised safety of care • Professional image in society
Pintanela de Carvalho et al. (2021) Relationship between workloads and presenteeism among nursing workers Australia	To analyze the relationship between exposure to workloads and presenteeism among nursing workers in the socio-environmental context of university hospitals	Cross-sectional study	372 nurses working in hospitals	**Organizational consequences** • There was a significant correlation between chemical loads and time management, biological loads and time management, physical demand and productivity loss, physiological loads and mental and interpersonal demand, production demand and loss of productivity, and psychological loads and production demand.
Rainbow (2019) Presenteeism: Nurse perceptions and consequences. United States	To describe factors that lead to and consequences of presenteeism	Qualitative research	295 nurses working in nursing organizations and hospitals	**Reasons for presenteeism** • In the case of each of these conditions, nurses put the needs of their hospital unit and co‐workers above their own health and often utilized coping strategies like caffeine, eating, regrouping, taking control when possible, and co‐worker and patient appreciation. • For some nurses, the importance of saving days away from work for upcoming planned trips or to take care of sick children meant they could not call in for their own illness. • For some nurses, it was the guilt of letting co‐workers down who would be working short, for others it was the guilt of letting patients down, and still for others it was guilt of letting family members down by not having available time off or receiving a smaller paycheck. **Consequences of presenteeism** • The participants described both proximal consequences, which alerted them to their decreased ability to perform, and distal consequences that radiated beyond the individual nurse, the result of the participant's changes in ability. • Proximal consequences were closer to the nurse experiencing them and included mental difficulties, and a worsened attitude when they were. • In addition to difficulty completing documentation, the nurses' mental state and attitude also impacted in‐person communication with co‐workers, family members, and patients. • The worsened attitude that nurses described was contagious and impacted the unit overall. • These consequences for the nurses' own health also could impact their families. • The consequences of nurse presenteeism span from workplace to personal to home.
Rainbow et al. (2020) Nurse health, work environment, presenteeism and patient safety United States	To explore the relationships between nurse fatigue, burnout, psychological well-being, team vitality, presenteeism, and patient safety	Cross-sectional study	332 nurses working in hospitals	**Influencing factors** • The model revealed significant negative relationships between job stress–related presenteeism and a lower frequency of events reported, perceived safety, and health care team vitality. • The model revealed significant positive relationships between burnout and the frequency of events reported, between psychological well-being and the frequency of events reported, and between health care team vitality and the frequency of events reported and perceived safety. • Psychological well-being and burnout were significantly and positively linked to job stress–related presenteeism. • In addition to job stress–related presenteeism, burnout was significantly positively linked to sickness presenteeism. • Job stress–related presenteeism completely mediated the relationship between psychological well-being and perceived safety, and the relationship between burnout and perceived safety. • Job stress–related presenteeism partially mediated the relationships between team vitality and perceived safety, between psychological well-being and the frequency of events reported, between burnout and the frequency of events reported, and between team vitality and the frequency of events reported.
Rainbow et al. (2021a) A qualitative study describing nurses' experiences with presenteeism United States	To understand nurse awareness of coping and decision making regarding presenteeism and the consequences	Qualitative research	16 nurses working in hospitals and medical centers	**Awareness and symptoms of presenteeism** • The participants often noticed that they were less than present when they described having a lack of focus, forgetfulness, sleepiness/fatigue, changes in mood, and, while they are sick, having symptoms of the illness. **Coping with presenteeism on and off shift** • The participants were able to describe both preventative and reactive coping strategies for addressing presenteeism. **Decision making regarding presenteeism** • The participants described considering staffing on the unit; workplace policies regarding calling in sick or leaving a shift early, including policies that involved potential disciplinary action for calling in sick; and stigma attached to calling in sick. **Consequences of presenteeism** • The subcategories of consequences that participants described included patients, team culture, and outside of work.
Rainbow et al. (2021b) Risky business: A mediated model of antecedents and consequences of presenteeism in nursing United States	To evaluate a model for presenteeism in nursing by examining the model's interrelationships and the fit of the data to the model.	Cross-sectional study	447 nurses working in hospitals	**Influencing factors** • Missed care was related to presenteeism and perceived stress. • Burnout was related to perceived stress and presenteeism. • Higher perceived stress, less work–life balance, and a positive work environment are related to presenteeism. **Consequences for nurses** • Directionality of the relationship suggests that higher presenteeism and perceived stress were both linked to increased burnout.
Santos et al. (2021) Factors associated with presenteeism in nursing workers Brazil	To analyze factors associated with presenteeism in nursing workers with sociodemographic variables, health and work conditions, productivity and musculoskeletal symptoms	Cross-sectional study		**Influencing factors** • Musculoskeletal complaints and shift work lead to productivity losses if people go to work despite complaints. **Consequences for nurses** • Reinforcement of musculoskeletal complaints **Organizational consequences** • Future absenteeism at work • Productivity loss of 8.8 % due to presenteeism
Shan et al. (2021) Presenteeism in nurses: Prevalence, consequences, and causes from the perspectives of nurses and chief nurses China	To evaluate the prevalence, consequences, and causes of presenteeism in Chinese nurses from the perspectives of nurses and chief nurses	Cross-sectional study	481 nurses and 282 chief nurses working in hospitals	**Prevalence of presenteeism** • In the previous 6 months, 394 nurses (94.25 %) experienced presenteeism. **Reasons for presenteeism** • Highest for organization related causes (51.44 %) • Workload (63.64 %) • Leave system (63.46 %), • Conscientiousness (59.10 %) • Financial needs (52.04 %) **Influencing factors** • Significant differences in nurse presenteeism scores in terms of the nurse's age, tenure, and marital status (p < 0.05) • The prevalence of nurse presenteeism increased significantly with increased age and tenure, and married nurses preferred to work while ill more than unmarried nurses. **Organizational consequences** • When working while ill, the average work efficiency was 74.08 %.
Shan et al. (2022) Authoritarian leadership and nurse presenteeism: the role of workload and leader identification China	To explore the impact and mechanism of authoritarian leadership on presenteeism	Cross-sectional study	528 nurses working in hospitals	**Influencing factors** • Authoritarian leadership influences presenteeism at all levels (model 5: γ = 0.24, p < 0.001) • Leader identification correlated negatively with workload (r = -0.23, p < 0.01) and presenteeism (r = -0.13, p < 0.01), while workload correlated positively with presenteeism (r = 0.47, p < 0.01). • More married nurses preferred to work while sick, compared with unmarried nurses (t = -2.26, p < 0.05). • *Post hoc* analysis indicated that the presenteeism of nurses with a bachelor's degree was significantly higher than that of nurses with lower educational levels.
Shan et al. (2023) Cross-level effects of health-promoting leadership on nurse presenteeism: The mediation and moderation effect of workload and performance pressure. China	To examine the relationship between health-promoting leadership and presenteeism in the context of leader-subordinate interaction	Cross-sectional study	98 chief nurses and 528 nurses working in hospitals	**Influencing factors** • Increased workload and health-promoting leadership leads to workload and stress leads to presenteeism among carers.
Shdaifat (2023) Presenteeism and productivity loss among nurses Saudi Arabia	To determine the productivity loss due to presenteeism from three dimensions and to evaluate the relationship between presenteeism rate and associated factors among nurses	Cross-sectional study	309 nurses working in hospitals	**Influencing factors** • Based on the health performance questionnaire, there was high absolute presenteeism among nurses who were aged over 32 years (mean 84.6, standard deviation 14.6, t(301)=-3.36, p = 0.001, d = 0.39), who had over a decade of work experience (mean 84.4, standard deviation 14.4, t(296) = -3.14, p = 0.002, d = 0.37), and who had one or more children (mean 83.3, standard deviation 14.5, t(300) = 2.37, p = 0.018, d = -0.28). • Nurses had a higher Stanford Presenteeism Scale score (high level of presenteeism) if they were older than age 32 years (mean 21.8, standard deviation 4.4, t(284) = -3.16, p = 0.002, d = 0.38), had >10 years of experience (mean 21.8, standard deviation 4.4, t(280) = -3.24, p = 0.001, d = 0.39), were married or widowed (F(2, 282) = 5.1, p = 0.007, ηp^2^ = 0.04), had children (mean 21.6, standard deviation 4.2, t(283) = 3.16, p = 0.002, d = -0.38), had two or three children (F(5, 274) = 2.89, p = 0.015, ηp^2^ = 0.05), and had obesity (F(2, 255) = 3.69, p = 0.013, ηp^2^ = 0.03). **Organizational consequences** • Productivity loss
Silva-Costa et al. (2020) Association between presenteeism, psychosocial aspects of work and common mental disorders among nursing personnel Brazil	To examine the mediating role of lost productivity from presenteeism (the ability to concentrate on work and to complete work despite a health problem) in the association between psychosocial factors at work (psychological demands, skills discretion, decision authority, and social support) and common mental disorders among nursing workers at a public hospital	Cross-sectional study	1218 nurses working in hospitals	**Prevalence of presenteeism** • 32.8 % **Influencing factors** • Presenteeism was more frequent among women, workers facing higher levels of psychological demands, those scoring higher in common mental disorder screening and those enjoying less social support.. Among the subgroup of presenteeist workers, common mental disorders correlated significantly (p < 0.050) and negatively with the ability to concentrate on work (r = -0.300), the ability to complete work despite a health problem (r = -0.153), psychosocial demands (r = 0.202), social support (r = -0.324), and decision authority (r = -0.108).
Simonetti et al. (2021) Presenteeism levels among Italian nurses. A multicentric survey Italy	To assess the prevalence of presenteeism among Italian nurses	Cross-sectional study	652 nurses	**Influencing factors** • Female sex seems to be a predictor of higher levels of presenteeism (p = 0.002), which also seems to increase, although slightly, among younger nurses (p = 0.040). • Presenteeism seems to be related to the presence of 2–4 self-reported health problems (p < 0.05). • Among nurses reporting ≥1 physical health problems, presenteeism was significantly higher among those reporting back pain and hypertension compared with those not affected by these conditions (p < 0.05).
Song et al. (2021) The effect of emotional labor on presenteeism of Chinese nurses in tertiary-level hospitals: The Mediating role of job burnout China	To evaluate the role of burnout as a mediating factor between the three types of emotional labor strategies and presenteeism among nurses in tertiary-level hospitals	Cross-sectional study	1038 nurses working in hospitals	**Influencing factors** • Presenteeism was explained by 22.8 % of the variance in the final model (p < 0.001). At the same time, more frequent weekly overtime and >50 years of age predicted higher presenteeism in the regression model. The results also revealed that the higher the score of emotional labor and burnout, the more frequent the presenteeism. • Presenteeism correlated positively with surface acting, emotionally expressed demands, deep acting, emotion exhaustion, depersonalization, and low personal accomplishment (p < 0.01).
Sousa et al. (2023) Common mental disorders, productivity and presenteeism in nursing workers Brazil	To investigate the association between the occurrence of common mental disorders with loss of productivity and presenteeism in nursing workers at a public health service	Cross-sectional study	458 nursing workers (127 nurses, 225 technicians, and 106 assistants) working in hospitals	**Prevalence of presenteeism** • 38.14 % **Influencing factors** • Workers with children • Diagnosed with depression • Migraine and mixed anxiety and depressive disorder • A history of sick leave in the last year (not observed when there was a leisure routine) • Workers with common mental disorders were four times more likely to experience presenteeism compared with those classified without the condition (odds ratio 4.27, 95 % confidence interval 2.43–7.67, p < 0.01).
Wu et al. (2024) The effect of occupational coping self-efficacy on presenteeism among ICU nurses in Chinese public hospitals: A cross-sectional study China	To investigate the current status of intensive care unit nurses’ occupational coping self-efficacy and presenteeism in public hospitals in western China and to explore the impact of intensive care unit nurses’ occupational coping self-efficacy on presenteeism	Cross-sectional study	722 nurses working in intensive care units	The presenteeism rate was 67.23 %. Correlation analysis showed that in intensive care unit nurses, the Occupational Coping Self-Efficacy Scale (OCSE-N) total score correlated negatively with the SPS-6 total score (r = −0.421, p < 0.05), indicating that the higher the level of occupational coping self-efficacy, the lower the presenteeism. The multiple hierarchical regression analysis showed that coping self-efficacy has a strong predictive power.
Yi et al. (2021) Health-related symptoms and working conditions on vulnerability to presenteeism among nurses in South Korea South Korea	To predict the characteristics of high-risk groups for presenteeism among nurses	Cross-sectional study	478 nurses working in hospitals	**Influencing factors** Presenteeism was significantly higher among those who experienced musculoskeletal pain, high emotional demands, discrimination, and psychological adverse social behavior at the workplace. The combined presence of psychological adverse social behavior and musculoskeletal pain predicted presenteeism; these should be treated as factors with a high risk of presenteeism.
Yoshimoto et al. (2020) Presenteeism and associated factors among nursing personnel with low back pain: A cross-sectional study. Japan	To identify the factors associated with presenteeism among nursing personnel with low back pain through the evaluation of several aspects, including individual, occupational, and psychological factors	Cross-sectional study	668 nurses working in medical centers	**Influencing factors** • The intensity of low back pain (p < 0.001), Tampa Scale for Kinesiophobia (TSK-11)(p < 0.001), Kessler Screening Scale for Psychological Distress (K6)(p < 0.001), workaholism (p < 0.001), overworking hours (p = 0.016), and frequency of shift work (p = 0.015) correlated positively with the degree of presenteeism.
Zhang et al. (2020) Nurses' job insecurity and emotional exhaustion: The mediating effect of presenteeism and the moderating effect of supervisor support China	To provide empirical evidence for prevention and intervention strategies that address nurses’ presenteeism behavior in the future	Cross-sectional study	330 nurses working in hospitals	**Influencing factors** • Job insecurity correlated positively with presenteeism (r = 0.24, p < 0.01) and emotional exhaustion (r = 0.22, p < 0.01). **Consequences for nurses** • Presenteeism behavior correlated positively with emotional exhaustion (r = 0.31, p < 0.01). Supervisor support correlated negatively with emotional exhaustion (r = -0.17, p < 0.01).
Zhang et al. (2024) Mediating effects of social support and presenteeism on turnover intention and post-traumatic stress disorder among Chinese nurses in the post-pandemic era: a cross-sectional study China	To investigate the relationship between turnover intention and post-traumatic stress disorder among Chinese nurses during post-pandemic era, and the mediating role of social support and presenteeism	Cross-sectional study	2513 nurses	Pearson correlation analyses showed that turnover intention correlated negatively with social support but positively with post-traumatic stress disorder and presenteeism.

## Appendix A JBI critical appraisal of the included studies

**Critical appraisal of the cross-sectional studies.**

**Critical appraisal of the qualitative studies.**

**Critical appraisal of the cohort Study**

**Critical appraisal of the reviews**

## Appendix B

**Summary of the included studies.**
